# Influence of Dosing Interval and Administration on the Bone Metabolism, Skeletal Effects, and Clinical Efficacy of Parathyroid Hormone in Treating Osteoporosis: A Narrative Review

**DOI:** 10.1002/jbm4.10005

**Published:** 2017-06-06

**Authors:** Kyoung Min Kim, Sae Young Lee, Yumie Rhee

**Affiliations:** ^1^ Division of Endocrinology and Metabolism Department of Internal Medicine Seoul National University Bundang Hospital and Seoul National University College of Medicine Seongnam Republic of Korea; ^2^ Lilly Korea Ltd. Jung‐ku Seoul Republic of Korea; ^3^ Department of Internal Medicine, Endocrine Research Institute Severance Hospital, Yonsei University College of Medicine Seoul Republic of Korea

**Keywords:** DOSING INTERVAL, FRACTURE REDUCTION, OSTEOPOROSIS, PARATHYROID HORMONE, TERIPARATIDE

## Abstract

Recombinant human parathyroid hormone (PTH) is the key anabolic agent used for preventing fracture in postmenopausal women with osteoporosis. In bone metabolism, PTH signaling is mediated through a G protein–coupled receptor that affects various post‐receptor signaling pathways. Results of preclinical and clinical studies have shown that PTH improves both the structure and strength of bone tissue. Once daily subcutaneous injection of the PTH fragment, teriparatide (PTH [1‐34]), is the most commonly recommended formulation and dosing strategy in clinical practice. However, other dosing intervals, formulations, and routes have been investigated in preclinical and clinical studies. In particular, once‐weekly and cyclical administration have been investigated mainly as a means of reducing the high direct costs of treatment. In preclinical studies, bone formation/resorption markers, bone mineral density measurements, and histomorphometric parameters improved with both once‐daily and once‐weekly administration. However, the magnitude and duration of such improvements were generally greater with once‐daily PTH administration. In clinical studies, reductions in fracture incidence were also noted with both once‐daily and once‐weekly PTH administration, although improvements in nonvertebral fractures are less evident with once‐weekly administration. This narrative review details the differences between PTH formulation and dosing strategies in relation to preclinical and clinical efficacy/safety parameters, although it should be stressed that no head‐to‐head studies allow direct comparisons. This review also seeks to outline practical considerations involved with PTH prescribing and new directions in research regarding routes of administration. © 2017 The Authors. *JBMR Plus* is published by Wiley Periodicals, Inc. on behalf of the American Society for Bone and Mineral Research.

## Introduction

Osteoporosis is a skeletal disorder characterized by decreased bone strength, which is determined by both bone quality (in terms of architecture, geometry, rate of turnover, damage accumulation, and degree of mineralization) and bone quantity, mostly represented by bone mineral density (BMD).^1^ Osteoporosis is characterized by thinning of cortical and cancellous bone with numerous resorption cavities acting as mechanical stress sites that increase the risk of fracture.^2^, ^3^, ^4^ The main pathophysiology of osteoporosis is caused by imbalanced bone remodeling. In young healthy adults, bone is renewed through continual and balanced resorption and formation, mainly via the effects of osteoclasts and osteoblasts, respectively.^5^ However, in older adults, especially during and after menopause in women, bone resorption exceeds formation leading to a net loss of bone, which can result in osteoporosis.^5^

Pharmacologic treatments for osteoporosis are broadly divided into antiresorptive and anabolic agents.^6^ Fundamentally, anabolic agents enhance bone formation, whereas antiresorptive agents reduce bone resorption. Mechanistically, anabolic agents increase new bone formation by increasing both osteoblast and osteoclast activity, which increases bone turnover and bone mass.^7^ Although both osteoblast and osteoclast activity are increased by anabolic agents, the precedent and great increase in bone formation with anabolic agents can be appreciated through an understanding of the anabolic window.^6^ According to this concept, the maximal rise in bone formation markers, particularly type 1 procollagen N‐terminal and C‐terminal propeptides (P1NP, P1CP), occurs early after anabolic agent administration, before a subsequent rise in bone resorption markers.^5^

In clinical practice, recombinant human PTH, which is available in two general forms, PTH (1‐34) and PTH (1‐84), is the most common form of anabolic therapy currently available. PTH (1‐34), or teriparatide, is an active, receptor‐binding fragment of PTH that is manufactured both as a recombinant fragment and as a synthetic product in the form of teriparatide acetate. The other form of PTH, PTH (1‐84), is a recombinant single‐chain, 84‐amino acid polypeptide identical to that of full‐length endogenous PTH.^8^ Recently, analogues of PTH‐related protein (PTHrP), an abundant factor in bone that shows N‐terminal homology with PTH, have also been investigated for osteoporosis treatment in preclinical and clinical studies.^9^ Both teriparatide and, to a lesser extent, PTH (1‐84) have been investigated in postmenopausal women with primary osteoporosis in large‐scale randomized trials and open‐label extension studies. Based on the findings of clinical trials, teriparatide is primarily indicated for the treatment of postmenopausal women with osteoporosis at high risk of fracture.^7^ Teriparatide is also indicated to treat men and women with osteoporosis associated with sustained systemic glucocorticoid therapy who are at high risk of fracture, and to increase bone mass in men with primary or hypogonadal osteoporosis who are at high risk of fracture.^7^ Although PTH (1‐84) has been investigated for treating osteoporosis, it is not approved for this indication, but is approved for the treatment of hypoparathyroidism and to control hypocalcemia in patients with hypoparathyroidism.^10^

Although other doses, dosing intervals, and routes of administration for both teriparatide and PTH (1‐84) have been investigated in preclinical and clinical studies, the current recommended dose of teriparatide in clinical practice is 20 μg once daily administered as a subcutaneous injection.^7^ Because once‐weekly PTH dosing involves higher doses, this dosing frequency is not approved for self‐administration. Cyclic dosing is similar to once‐daily administration in terms of dose and frequency, except each treatment cycle is followed by a similar length cycle without PTH, and is usually covered with bisphosphonate therapy. These different dosing strategies may influence the efficacy, safety, adherence, and cost of treatment, although no reviews to date have summarized or compared these strategies.

The aim of this review is to summarize and compare different strategies for PTH dosing in terms of interval and route of administration with reference to mechanism of action, clinical efficacy, and safety. In practical terms, this review can help clinicians who care for osteoporosis patients at high risk of fracture, understand the differences between different dosing intervals or administration routes of PTH and, thereby, make appropriate prescribing decisions.

## Overview of Parathyroid Hormone Signaling in Bone Metabolism

**Figure 1:**
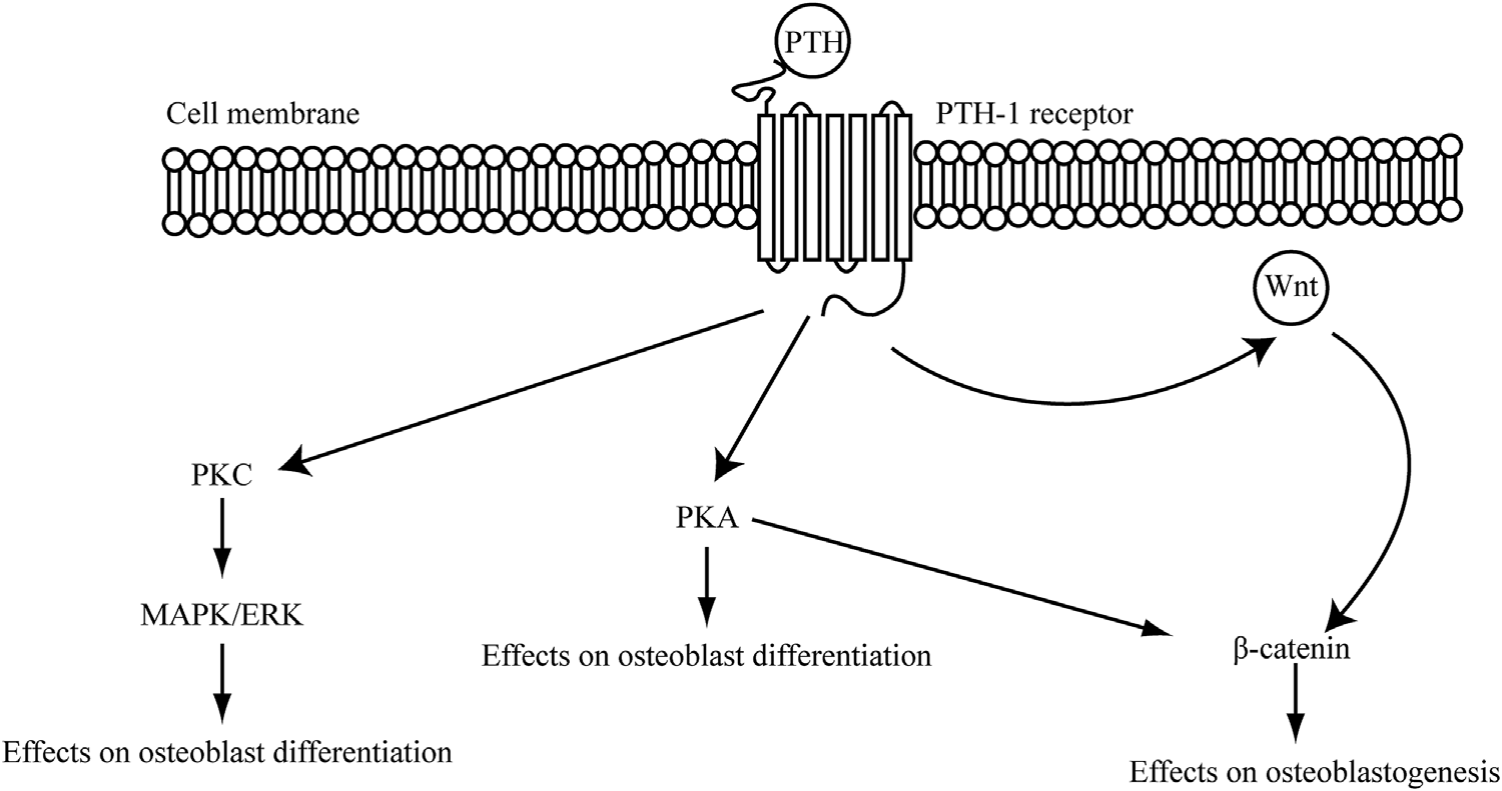
Principal mechanisms of parathyroid hormone signaling in bone. The Wnt signaling pathway is essential in many biological processes. ERK = extracellular signal‐regulated kinases; MAPK = mitogen‐activated protein kinases; PKA = phosphokinase A; PKC = phosphokinase C; PTH = parathyroid hormone; Wnt = Wingless‐type MMTV integration site family member.

The PTH‐1 receptor is a G protein‐coupled receptor (GPCR) that mediates most of the functions of both PTH and PTHrP.^(6,11)^ During initial PTH‐1 receptor activation, the C‐terminal and N‐terminal of the ligand interact with the N‐terminal region and transmembrane regions of the receptor, respectively.^11^ Subsequent signal transduction is mediated by receptor phosphorylation via G receptor kinases, recruitment of β‐arrestin proteins, and receptor internalization. This, in turn, leads to activation of both the cyclic adenosine monophosphate (cAMP)‐dependent protein kinase A and calcium‐dependent protein kinase C signaling pathways, which regulate osteoblast function (Fig. 1).^6^, ^11^ Several other pathways, including the ubiquitous mitogen‐activated protein (MAP) kinase and phospholipase A and D pathways have also been implicated in signaling.^6^, ^12^ Finally, PTH signal propagation may lead to regulation of transcription of key genes involved in the balance of osteoblastic and osteoclastic activity. Among these, PTH may activate the Wnt/β‐catenin pathway via, for example, downregulation of the expression of the Wnt antagonist sclerostin.^6^, ^13^ This provides a favorable balance toward osteoblastogenesis, as sclerostin acts as a potent inhibitor of this process.^14^, ^15^

## Molecular Mechanisms and Action in Bone Metabolism

As noted, PTH controls calcium homeostasis via interaction with PTH receptors expressed on osteoblasts, osteocytes, bone lining, and other cell types.^(16)^ The relative action of PTH on osteoblasts and osteoclasts, and hence its skeletal effects, appears to be largely determined by the pattern of systemic exposure. Following administration of PTH, bone formation markers increase rapidly followed by a lesser and delayed increase in resorption markers.^16^ This differential response underlies the concept of the anabolic window, whereby intermittent administration causes a relative increase in osteoblastic activity compared with osteoclastic activity, leading to a net increase in bone with each cycle.^17^, ^18^ Studies in animals using gene expression and molecular endpoints with different administration protocols support this concept. Human PTH (1‐34) administered daily to mice led to a decrease in osteoblast apoptosis and an increase in osteoblastic indices, such as osteoblast perimeter.^19^ A study of rats similarly found that daily PTH administration led to a dramatic increase in osteoblast numbers and corresponding increases in osteoblastic indices.^20^ On the other hand, continuous PTH administration has been shown to lead to an increase in osteoclastic activity. Rats receiving a continuous infusion of PTH (1‐38) for 1 to 24 hours had a dose‐dependent increase in osteoclast number that was associated with increased nuclear factor κB ligand (RANKL) mRNA expression and decreased expression of osteoprotegerin mRNA expression.^(21)^ PTH also inhibits bone morphogenetic protein expression, which may contribute to the waning of the anabolic response that is observed with continuous treatment.^22^

In addition to changes in genetic and molecular endpoints, preclinical and clinical studies using histomorphometric analyses have shown that teriparatide increases cancellous bone volume, bone mass, and cortical width, and improves bone architecture (eg, increased connectivity of split trabecula and increased trabecular number, without a significant change in cancellous bone volume).^16^, ^23^, ^24^, ^25^ However, animal studies reveal that bone remodeling with PTH treatment increases intracortical bone turnover and porosity, mostly near the endocortical surface.^21^, ^24^ This increase in cortical porosity did not interfere with an increase in bone strength, with one study showing improvements in bone mass and improved structural architecture, including increased cortical width and trabecular volume/number.^24^

## Effect of PTH Dosing Interval on Mechanistic and Clinical Parameters

Preclinical and clinical studies of both PTH (1‐84) and teriparatide have used a variety of dosing intervals across a range of treatment durations. The most commonly assessed dosing intervals include once‐daily, cyclical (once‐daily with treatment rest periods), and once‐weekly dosing. Throughout these clinical studies, assessments were broadly made in terms of (1) bone formation markers, (2) BMD, (3) histomorphometric analyses, and (4) clinical fractures.

### Bone turnover markers

Serologic bone turnover markers, especially bone formation markers, have been used to monitor the efficacy of teriparatide and PTH. The response of bone turnover markers significantly relates to both the mechanisms of action of PTH on bone and clinical effects, as commonly assessed by measures such as BMD.

#### Preclinical studies

Preclinical studies have directly compared the effects of different teriparatide dosing intervals on bone formation markers. In a comparative study of female mice (*n* = 7/group), both teriparatide 40 μg/kg/day once daily over 7 weeks and teriparatide 40 μg/kg/day once daily alternating once weekly with vehicle for 7 weeks (cyclic) significantly increased osteocalcin (once daily, 330%; cyclic, 260%).^26^ Both once‐daily and cyclic teriparatide administration increased BMD, although the increase was greater with once‐daily administration (16% to 17% at all sites) compared with cyclic administration (9% to 12% at all sites).^26^

#### Clinical studies

In clinical studies, changes in common bone formation and resorption markers were assessed in response to teriparatide and PTH (1‐84) administered at once‐daily, cyclic, and once‐weekly dosing intervals (Table 1).^27^, ^28^, ^29^, ^30^, ^31^, ^32^, ^33^, ^34^, ^35^, ^36^, ^37^ In general, rapid increases in bone formation markers such as alkaline phosphatase (ALP) and P1NP within 1 to 3 months were reported in these studies.^27^, ^28^, ^29^, ^30^, ^33^, ^34^ Increases in bone formation markers were greater with more frequent administration and higher doses of teriparatide (regardless of the dosing interval).^27^, ^32^ The increase in bone formation markers was also characterized by a gradual slowing in the rate of increase with prolonged therapy and eventual decline following cessation of treatment. However, at the end of treatment, bone formation markers remained elevated above baseline levels for once‐daily administration of teriparatide, whereas they generally returned to baseline levels with once‐weekly administration (Table 1). In several of these studies, the rapid rise in bone formation markers was accompanied by a delayed and more gradual rise in bone resorption markers such as type I collagen cross‐linked N‐telopeptide.^28^, ^30^, ^34^, ^36^, ^38^

**Table 1:** Initial and End‐of‐Treatment Response of Common Bone Turnover markers to Various PTH Agents and Dosing Intervals

				Bone marker response in PTH‐treated patients initially (and at end of treatment)					
				Formation				Resorption	
Reference	Design	Treatment (*n*)	Duration (months)	ALP	P1NP	P1CP	OC	NTX	Comment
Once‐daily									
Chen and colleagues^(27)^ (2005)	RCT, PC	Placebo (175); Teri 20 μg (171); Teri 40 μg (174)	19 (36 planned)	↑ (↑)	↑ (↑)	↑ (↑)		↑ (↑)	Increases were dose‐dependent
Cosman and colleagues^(28)^ (2005)	RCT	Teri 25 μg^a^ (43)	15	↑ (↑)	↑ (↑)		↑ (↑)		Greatest and most rapid increase seen in P1NP
Cosman and colleagues^(29)^ (2015)	RCT, OL	Teri 20 μg^c^ (73)	24		↑ (↑)		↑ (↑)		Greater increases in alendronate‐naive patients
Dobnig and colleagues^(30)^ (2005)	RCT, DB	Teri 20/40 μg (36); placebo (21)	20^b^	↑ (↑)		↑ (↑)		↑ (↑)	ALP and PICP increased rapidly
Greenspan and colleagues^(31)^ (2007)	RCT, DB, PC	PTH (1‐84) 100 μg (1286); placebo (1246)	18	↑ (↑)				↑ (↑)	ALP increased in 1 month; NTX in 6–12 months
Horwitz and colleagues^(36)^ (2013)	RCT	Teri 20 μg (35); PTH (1‐36) 400 μg (35); PTH (1‐36) 600 μg (35)	3		↑ (↑)				Increase in P1NP in Teri > PTH (1‐36)
Niimi and colleagues^37^ (2015)	Observational	Teri 20 μg (381)	12		↑ (↑)			↑ (↑)	Absolute increase in P1NP was similar for men and women; absolute increase in NTX was lower for men than women
Orwoll and colleagues^32^ (2003)	RCT, DB, PC	Placebo (147); Teri 20 μg (151); Teri 40 μg (139)	11^b^	↑ (↑)		↑ (↓)		↑ (↑)	Increases were dose‐dependent
Cyclic									
Cosman and colleagues^28^ (2005)	RCT	Teri 25 μg^a^ (43)	15	↑ (↑)	↑ (↑)		↑ (↑)		Marker levels decreased during “off” periods; peak levels lower than for once‐daily administration
Cosman and colleagues^29^ (2015)	RCT, OL	Teri 20 μg^c^ (73)	24		↑ (↑)		↑ (↑)		
Once‐weekly									
Black and colleagues^33^ (2008)	RCT, DB, PC	Placebo (25); PTH (1‐84) 100 μg (25)	12		↑ (↓)				Rapid rise and gradual decline in P1NP over treatment period
Nakamura and colleagues^34^ (2012)	RCT, DB, PC	Placebo (286); Teri 56.5 μg (286)	18		↑ (↓)		↑ (−)	↑ (↓)	P1NP and OC levels decreased after initial marked rise
Tanaka and colleagues^35^ (2014)	Analysis of RCT, DB, PC	Placebo (130); Teri 56.5 μg (107)	18	↓ (↓)	↑ (↓)		↑ (↓)	↑ (↑)	P1NP and OC levels decreased after initial marked rise

No direct comparative studies have been published to compare dosing strategies; down arrow indicates decrease from previous increased levels to baseline or above baseline levels.

PTH = parathyroid hormone; ALP = alkaline phosphatase; P1NP = procollagen N‐terminal type 1 propeptide; P1CP = procollagen C‐terminal type 1 propeptide; OC = osteocalcin; NTX = type I collagen cross‐linked N‐telopeptide; RCT = randomized controlled trial; PC = placebo‐controlled; Teri = teriparatide; OL = open‐label; DB = double‐blind.

^a^ Plus alendronate.

^b^ Median.

^c^ With or without alendronate.

In a small number of clinical studies, cyclic PTH administration has been shown to be similarly effective to daily administration on a number of key indices.^28^, ^29^ In one randomized study of 126 women with osteoporosis receiving alendronate, both daily continuous PTH (“daily” administration) and daily PTH for three 3‐month cycles alternating with 3‐month periods without PTH (“cyclic” administration) led to rapid increases in bone formation indexes, although bone formation declined during cycles without PTH in the cyclic group.^28^ Importantly, spinal BMD rose significantly in both groups (6.1% daily‐treatment group, 5.4% in the cyclic‐therapy group; *p* = NS for the difference between PTH groups). A more recent study using a similar protocol of daily PTH or intermittent 3‐month cyclic administration found that, among alendronate‐naive patients, BMD increased significantly in the lumbar spine, hip, and trochanter in both daily and cyclic groups although increases were greater in the daily group. In alendronate‐treated women, improvements in BMD were similar between the daily and cyclic groups, despite the later receiving only 50% of the PTH dose.^29^

#### Teriparatide

Once‐daily teriparatide administration generally leads to a rapid rise in bone turnover markers.^27^, ^30^, ^32^, ^36^, ^39^ One open‐label study of once‐daily teriparatide treatment in 15 postmenopausal women with osteopenia found that P1NP levels increased rapidly by 111% above baseline levels after 28 days.^39^ P1CP and osteocalcin also showed a similar, but less pronounced, pattern of increase.^39^ Such early and rapid changes in P1NP have been shown to correlate both positively and significantly with later BMD response at 18 to 24 months.^27^, ^40^ For example, in an analysis of an open‐label, 2‐year clinical trial of teriparatide in postmenopausal women with severe osteoporosis (European Study of Forsteo [EUROFOS]), the greatest correlation was noted between P1NP concentration at 1 month and the change in lumbar spine BMD to 24 months (*r* = 0.365; *p* < 0.0001).^40^ In the Fracture Prevention Trial involving postmenopausal women with osteoporosis, once‐daily treatment with teriparatide led to increases in P1NP at 3 months that were highly correlated with increases in lumbar spine BMD at 18 months (Spearman correlation coefficient = 0.62, *p* < 0.05).^27^ Other individual studies highlight the rapid and significant rise in osteoblastic markers, especially P1NP, with once‐daily teriparatide administration. In two randomized controlled trials among women with osteoporosis, P1NP increased by 310% at 6 months and 373% at 15 months following once‐daily teriparatide 20 μg and 25 μg, respectively.^28^, ^29^ A retrospective investigation of 488 women who received once‐daily teriparatide 20 μg reported specific values showing that P1NP levels rose from a baseline value of 52.9 ± 39.1 μg/L to 159.1 ± 118.3 μg/L at 4 months, after which levels declined to 128.9 ± 88.7 μg/L at 12 months.^37^ As mentioned, increases in bone turnover markers tended to be greater with higher teriparatide doses. For example, in one randomized controlled trial of teriparatide treatment in 437 men with reduced spine or hip BMD, the median percent change from baseline in P1CP was approximately 30% with teriparatide 20 μg once daily versus approximately 80% with teriparatide 40 μg once daily after 1 month of treatment.^32^

Markers of bone formation in patients receiving once‐daily teriparatide generally remained elevated for the duration of treatment (Table 1). For example, in the Fracture Prevention Trial, increases in ALP, P1NP, and P1CP compared to baseline were significantly higher versus placebo from 3 months to the end of treatment (12 months).^27^ Similarly, the levels of ALP, P1NP, and osteocalcin in women receiving once‐daily teriparatide 20 μg steadily rose during treatment and remained elevated after 15 months.^28^

The rise in markers of bone formation with once‐weekly teriparatide administration does not appear to be sustained for the duration of treatment.^33^, ^34^, ^35^ Findings from two randomized controlled trials demonstrated that P1NP increased by approximately 15% to 20% above baseline levels after 1 month.^34^, ^35^ However, in one of these trials, the positive change from baseline in P1NP began to decrease after 4 weeks and continued to decline to below baseline levels for the duration of treatment.^34^ As such, these clinical studies confirmed a lower increase in bone markers compared with once‐daily administration as well as the differential increase in bone formation compared with bone resorption with PTH treatment, consistent with the concept of an anabolic window. Moreover, increases in bone formation markers tended to be greater in treatment‐naive patients compared with those who had received bisphosphonate (eg, alendronate) therapy.^29^

#### PTH (1‐84)

Results related to bone turnover markers from a smaller set of studies of PTH (1‐84) largely reflect those seen with teriparatide.^31^, ^33^ In a randomized, double‐blind, placebo‐controlled trial of PTH (1‐84) in postmenopausal women, results from a subset of 600 enrolled patients showed that ALP peaked at 6 to 12 months (>80% change from baseline) and remained 60% higher at 18 months.^31^ In another randomized, placebo‐controlled trial, postmenopausal women received once‐daily PTH (1‐84) 100 μg or placebo for 1 month, followed by once‐weekly PTH (1‐84) or placebo for 11 months.^(33)^ Among women who received PTH (1‐84), P1NP increased by 98% versus baseline levels at 1 month and remained significantly higher than placebo at 3 and 6 months (*p* < 0.01).^33^

### BMD

**Figure 2:**
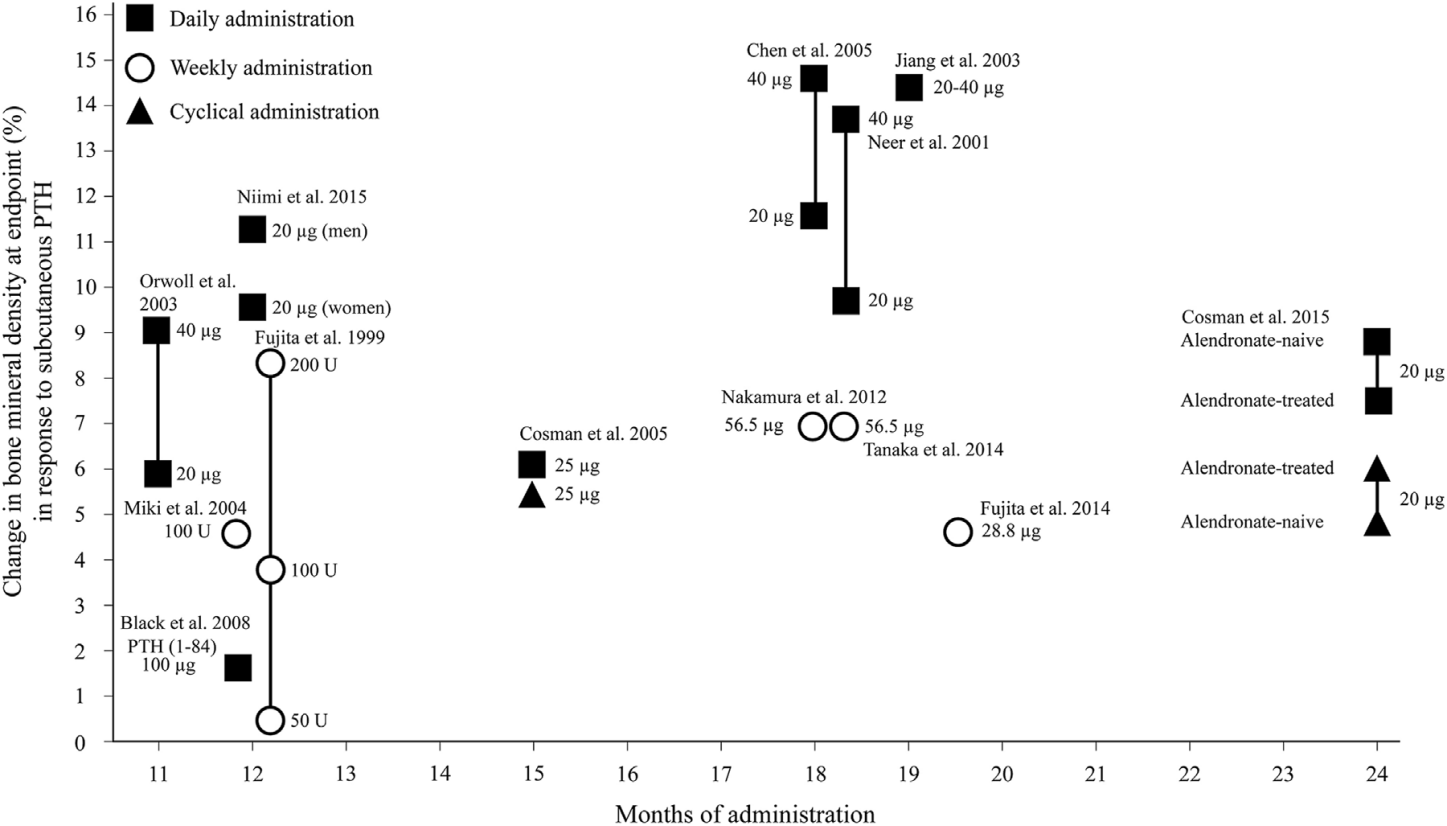
Change in bone mineral density in lumbar spine at endpoint (%) in response to subcutaneous teriparatide (unless otherwise states) at different doses for once‐daily and once‐weekly administration. Note that no direct comparative studies have been published to compare dosing strategies. PTH = parathyroid hormone; U = units.

BMD is a frequently used proxy measure of overall bone strength and, based on epidemiological studies, is also a strong predictor of fracture risk.^(1)^ As such, BMD represents a useful measure to link the skeletal and clinical effects of PTH. Clinical studies have also demonstrated both once‐daily and once‐weekly administration of the various formulations of PTH increases BMD (Fig.2).^25^, ^27^, ^28^, ^29^, ^33^, ^34^, ^35^, ^37^, ^41^, ^42^, ^43^, ^44^

#### Teriparatide

Across clinical studies of teriparatide, increases in BMD tend to be dose‐dependent and also greater with longer duration of treatment (Fig. 2). Although there are no direct comparisons between once‐daily and once‐weekly teriparatide administration, the results of various studies suggest that the magnitude of increases in BMD are greater in patients receiving once‐daily administration.

Several large randomized controlled trials provide an indication of the potential extent of the improvement in BMD with once‐daily teriparatide administration. In the Fracture Prevention Trial, the change in lumbar BMD at 18 months was 11.6% ± 7.4% for teriparatide 20 μg and 14.6% ± 8.6% for teriparatide 40 μg.^27^ Another randomized trial of 1637 postmenopausal women reported similar levels of change in lumbar BMD with once‐daily teriparatide at approximately 18 months (9.7% ± 7.4% for teriparatide 20 μg and 13.7% ± 9.7% for teriparatide 40 μg).^41^ Similar, albeit numerically smaller, improvements were also noted in the change in BMD of the femoral neck in these studies.

With once‐weekly teriparatide administration, improvements in BMD were also noted although these tended to be numerically lower compared with once‐daily administration (Fig. 2). For example, in two randomized controlled trials of once‐weekly teriparatide administration, the change in lumbar BMD at 18 months was 4.4% ± 4.7% for teriparatide 28.2 μg (*n* = 150) and 6.7% for teriparatide 56.5 μg (*n* = 286), respectively.^(34,42)^

#### PTH (1‐84)

A small number of clinical studies also reveal significant improvements in BMD following PTH (1‐84) administration. In a randomized, placebo‐controlled trial of postmenopausal women treated with once‐weekly PTH (1‐84) 100 μg, a mean percentage change of 6.53% in BMD compared with placebo was seen among 859 women (*p* < 0.001).^31^ In another randomized, placebo‐controlled trial, postmenopausal women treated with once‐weekly PTH (1‐84) 100 μg, vertebral trabecular BMD increased by 3.8% compared with placebo‐treated women (*p* = 0.08).^33^

### Histomorphometric parameters

Common structural indices used in studies of PTH include trabecular number and thickness for cancellous bone, and cortical bone ratio and thickness for cortical bone structure.^43^, ^44^ Common derived dynamic indices in clinical studies include mineralizing surface to assess the extent of surface active in mineralization at a particular time, mineral apposition rate, and bone formation rate.^43^, ^45^, ^46^ It should be noted that, in postmenopausal osteoporosis, bone formation rate increases as a compensatory response to the increased rate of bone resorption, but formation rates do not match the resorption rates with the net effect of bone loss.^46^ Iliac crest bone biopsies can also help establish the mechanism of action of PTH through determination of key two‐dimensional (2D) and three‐dimensional (3D) histomorphometric parameters related to bone turnover, mineralization, and bone volume.^43^, ^46^, ^47^

Regarding clinical studies, histomorphometric analyses from studies of once‐daily and once‐weekly administration have been conducted with different forms and formulations of PTH, using different methodologies and included parameters. These differences make it challenging to compare studies directly, although it appears that different dosing strategies lead to increases in bone volume and thickness, especially in cancellous bone, and other improvements in structural indices.

#### Teriparatide

Several studies have confirmed the benefits of once‐daily teriparatide administration in terms of skeletal parameters.^25^, ^30^, ^48^ A substudy of postmenopausal women with osteoporosis enrolled in the Fracture Prevention Trial showed that teriparatide 20 to 40 μg once daily significantly increased cancellous bone volume and connectivity, improved cancellous bone morphology with a shift toward a more plate‐like structure, and increased cortical bone thickness compared with placebo.^25^ Further, in a randomized, multicenter, double‐blind, placebo‐controlled trial, teriparatide 20 μg or 40 μg once daily led to improvements at 22 months in wall thickness, cancellous bone volume, marrow star volume, 3D trabecular thickness, and the ratio of bone volume to cancellous bone volume.^30^ However, in a randomized, open‐label study of postmenopausal women with osteoporosis, some differences were noted with once‐daily or cyclic teriparatide when added to ongoing alendronate 70 mg/week.^48^ Teriparatide led to significant increases in bone formation indices for cancellous and cortical bone although, in alendronate‐naive patients, the bone formation rate was higher in patients receiving once‐daily teriparatide compared with those receiving cyclic teriparatide.^48^ In contrast, the bone formation rate was similar with once‐daily and cyclic administration in patients who had received prior alendronate. Compared with zoledronic acid, teriparatide has been shown to lead to significantly higher values of mineralizing surface/bone surface and bone formation rate/bone surface indices as well as other differences in dynamic and static histomorphometric indices.^45^ These distinctions are based on the fundamental differences in mechanism of action between teriparatide, which acts as an anabolic agent with osteoblastic activity, and the antiresorptive agent with inhibitory effects on osteoclasts.

Regarding once‐weekly teriparatide administration, several clinical studies provide some evidence of positive histomorphometric benefits in terms of static and dynamic indices.^33^, ^49^ An observational study of 10 patients administered once‐weekly teriparatide 100 units (30 μg) for 1 year revealed an increase in bone volume, osteoid surface, and a tendency for an increase in other parameters of bone formation, plus improvement in cancellous bone continuity.^49^ However, the small patient numbers and lack of statistical significance associated with most of these results limit their interpretation.

#### PTH (1‐84)

Regarding PTH (1‐84), a double‐blind, randomized, placebo‐controlled trial of 50 postmenopausal women found that those who received PTH (1‐84) 100 μg once weekly had higher distal radial cancellous bone volume and trabecular number and thickness according to MRI assessment compared with placebo‐treated women (*p* < 0.04).^33^ Similarly, another randomized, placebo‐controlled trial found that postmenopausal women treated with once‐weekly PTH (1‐84) 100 μg had statistically significant differences in cortical bone volume and cortical volumetric bone mineral content compared with women treated with placebo.^31^

### Fracture prevention

In clinical studies of teriparatide and PTH (1‐84) treatment, fracture prevention has been mainly assessed by measures of fracture risk reduction and absolute numbers of fractures.

#### Teriparatide

Several studies have shown that once‐daily teriparatide has been associated with lower rates of vertebral and nonvertebral fractures compared with placebo or other agents.^28^, ^32^, ^41^ In a large, randomized trial of 1637 postmenopausal women with prior vertebral fractures, teriparatide 20 μg and 40 μg reduced the risk of new vertebral fractures versus placebo by 65% and 69% and nonvertebral fragility fractures versus placebo by 53% and 54%, respectively.^41^ In this study, there was a reduction in the total number of vertebral fractures of 136 per 1000 patient‐years in the placebo group, 49 per 1000 patient‐years in the teriparatide 20 μg group, and 30 per 1000 patient‐years in the teriparatide 40 μg group.^41^ Another randomized trial of 126 women with osteoporosis treated with alendronate for more than 1 year found that new or worsening vertebral deformities occurred in 1 of 38 women (3%) given once‐daily PTH (1‐34), 2 of 34 women (6%) given cyclic PTH (1‐34), and 4 of 36 women (11%) treated with alendronate (*p* = 0.20 for the difference among the groups).^28^

Once‐weekly teriparatide has been shown to reduce the risk of vertebral but not nonvertebral fractures versus placebo in several key clinical studies.^34^, ^42^, ^50^ In one randomized trial of 316 patients with osteoporosis, incident vertebral fractures occurred in 3.3% of patients who received teriparatide 28.2 μg once weekly compared with 12.6% of placebo‐treated patients over 78 weeks.^42^ Significant reductions in the risk of vertebral fracture were also observed between the two groups during the first 26 and 52 weeks.^42^ No significant differences between teriparatide and placebo were observed in the incidence of nonvertebral fragility fractures in this study. Further, in a randomized trial of 578 patients with prevalent vertebral fracture in the Teriparatide Once‐Weekly Efficacy Research (TOWER) trial, new vertebral fractures were less frequent in patients who received teriparatide 56.5 μg (3.1%) compared with those who received placebo (14.5%; *p* < 0.01); data on nonvertebral fractures were not reported in this study.^34^ A subgroup analysis of the TOWER trial also found that the overall relative risk (RR) of incident vertebral fracture between once‐weekly teriparatide‐treated and placebo‐treated patients was 0.20; data on nonvertebral fractures were not available in the TOWER trial.^(50)^ Teriparatide also significantly reduced the RR of incident vertebral fracture in both younger (<75 years, RR = 0.06, *p* = 0.007) and older (≥75 years, RR = 0.32, *p* = 0.015) patients.^50^ Overall, although there are no direct comparisons between once‐daily and once‐weekly teriparatide treatment, the results of these clinical studies suggest that fracture prevention is achievable with both dosing strategies.

#### PTH (1‐84)

Among the studies analyzed for this review, one randomized trial showed that PTH (1‐84) also led to significant reductions in the rate of fractures among postmenopausal women.^31^ In this placebo‐controlled trial using PTH (1‐84) 100 μg once daily, the overall incidence of new or worsened vertebral fractures was significantly reduced in patients who received PTH (1‐84) compared with those who received placebo (1.4% versus 3.4%, *p* = 0.001).^31^ Further, the incidence of new vertebral fractures was also reduced in PTH (1‐84)‐treated patients among those with no baseline fracture (0.7% versus 2.1%, *p* = 0.006) and among those with baseline fractures (4.2% versus 8.9 %, *p* = 0.04). The authors concluded that these results demonstrated that PTH (1‐84) seems to be effective for reducing the risk of new or worsened vertebral fractures in postmenopausal women with or without previous vertebral fracture.

### Safety and tolerability

Adverse events (AEs) and key laboratory abnormalities have been recorded in most clinical studies using both once‐daily and once‐weekly teriparatide. In placebo‐controlled studies of once‐daily teriparatide, rates of AEs were generally similar between teriparatide and placebo groups.^29^, ^32^, ^41^ However, higher rates of headache and nausea were reported with higher doses of teriparatide in one comparative trial.^32^ Compared with cyclic therapy, once‐daily administration of teriparatide was associated with a significantly higher percentage of musculoskeletal symptoms (26% versus 12%; *p* < 0.05), but a lower percentage of injection site redness (3% versus 18%; *p* < 0.05).^28^ The most common laboratory abnormality noted was hypercalcemia, although this was generally mild and occurred early in the course of treatment.^28^, ^41^

In clinical studies of once‐weekly teriparatide, rates of AEs were also generally similar between treatment groups, although there was a tendency toward greater rates of AEs and hypercalcemia with teriparatide, especially at higher doses.^34^, ^42^, ^51^ Among patients who received low‐dose (28.2 μg) teriparatide once weekly in one randomized, placebo‐controlled trial, there was a greater total incidence of adverse drug reactions (mainly nausea [5.7%] and vomiting [3.8%]) in patients who received teriparatide (24.1%) than those who received placebo (9.5%).^42^ In an open‐label trial of three dose levels of teriparatide once weekly, AEs (particularly nausea and headache) and abnormal laboratory findings were more frequent among patients who received the highest dose level (200 units weekly).^51^ In the TOWER trial, the overall incidence of AEs was similar between patients who received teriparatide once weekly and those who received placebo.^34^ However, there was a higher incidence of nausea, vomiting, headache, and abdominal discomfort among patients who received teriparatide, and the discontinuation rate was significantly higher in the once‐weekly teriparatide group.^34^

The finding of a high rate of sarcoma in rats receiving teriparatide at the highest‐tested dose level led to a black box warning in the United States for patients at increased baseline risk for osteosarcoma (eg, those with Paget's disease of bone or unexplained elevations of ALP).^7^, ^52^ The risk among rats appeared to be primarily dependent on the dose and duration of treatment, with the greatest risk found with treatment of 20 to 24 months (>70% of expected lifespan of rats).^53^ However, among humans, a causal association between teriparatide and osteosarcoma has not been established.^54^

### Novel formulations

Novel formulations of teriparatide, including intranasal, transdermal, and oral formulations, have been developed and tested in both preclinical and clinical studies. For example, a preclinical study in rats found that intranasal teriparatide was rapidly absorbed and produced maximum plasma concentrations within approximately 15 min with no apparent adverse effect on nasal epithelial integrity.^55^ More recently, another study in rats found that intranasal teriparatide significantly increased vertebral BMD compared with intranasal saline.^56^ A pilot randomized, open‐label, clinical trial showed that once‐daily nasal teriparatide at doses of 250 μg, 500 μg, or 1000 μg for 3 months increased lumbar BMD in a dose‐dependent manner, although only the 1000‐μg dose produced consistent and statistically significant changes in markers of bone turnover.^57^ In this study, an increase in bone formation markers (P1NP and osteocalcin) from baseline was also noted.^57^ A transdermal patch formulation of teriparatide, providing a rapid, pulse delivery has been assessed in a randomized, placebo‐controlled trial among 165 postmenopausal women with osteoporosis.^58^ In this trial, a teriparatide patch (20 μg, 30 μg, or 40 μg) or a placebo patch was self‐administered daily for 30 min, or teriparatide 20 μg was injected daily. The teriparatide transdermal patch significantly increased lumbar spine BMD versus placebo in a dose‐dependent manner at 6 months (*p* < 0.001) and the highest patch dose (40 μg) increased total hip BMD compared with both placebo patch and teriparatide injection. Further, bone turnover markers, including P1NP, increased in a dose‐dependent manner in all treatment groups. These results suggest that transdermal teriparatide delivery in postmenopausal women with osteoporosis is a potential alternative to daily subcutaneous injections.

Overall, novel formulations of teriparatide appear to be tolerable and may be clinically effective, although further studies are required to validate the clinical efficacy of these new methods of administration. Such new formulations may also have the potential to improve adherence by eliminating the need for subcutaneous injection.

## Implications for Clinical Practice

Based on the results of preclinical and clinical studies, once‐daily, cyclic, and once‐weekly teriparatide and PTH (1‐84) appear to have effects on skeletal parameters and fracture reduction. However, the different pattern of change in bone turnover markers implies that there could be some differences in bone metabolism at different dosing intervals. Increases in bone formation markers and BMD appear to be greater and more sustained with once‐daily teriparatide and PTH (1‐84) than with once‐weekly administration. Further, greater increases in these parameters have been associated with higher overall doses of PTH. Both these observations are consistent with an association between effect and overall intensity of treatment. Improvements in histomorphometric parameters consistent with the mechanism of action of teriparatide have been noted in preclinical and clinical studies of once‐daily administration. However, further data are required in clinical studies of once‐weekly administration to clearly establish these improvements. Differences in methodology, especially in relation to outcome measures, also make it challenging to compare results of these studies across different dosing intervals. Clinical studies involving head‐to‐head comparisons between dosing intervals would help clarify this issue.

Despite apparent differences in skeletal parameters with dosing intervals, improvements in clinical efficacy (eg, in terms of fracture reduction or risk of refracture) have been noted with both once‐daily and once‐weekly teriparatide. Tolerability and laboratory abnormalities noted in clinical trials involving various dosing strategies are generally consistent with known effects of PTH. There is some suggestion based on clinical study results that once‐weekly administration causes slightly higher rates of nausea compared with once‐daily administration, which might be caused by higher rates of hypercalcemia associated with the higher once‐weekly dose requirement. Overall, these results suggest that both once‐daily and once‐weekly teriparatide are clinically effective and well tolerated, despite some possible differences in effects directly related to mechanism of action, such as differences in the extent and rate of rise in bone formation markers and BMD.

As evidenced by clinical trials, most patients with osteoporosis treated with PTH respond well and the rate of persistence with treatment has also been noted to be high.^59^ However, a relatively small proportion (possibly around 10%) of patients with osteoporosis do not respond to teriparatide treatment.^60^ Long‐term retrospective analyses have helped to define the predictors of nonresponsiveness.^60^, ^61^ In one analysis of adults with osteopenia or osteoporosis treated with teriparatide at the Mayo Clinic, prior bisphosphonate treatment and vitamin D therapy were significantly associated with teriparatide treatment failure.^61^ A similar analysis conducted in Japan, prior bisphosphonate use, a lower baseline P1NP concentration, and a lower urinary N‐terminal telopeptide concentration at baseline were significantly associated with nonresponsiveness to teriparatide.^60^ An apparent lower BMD response at the hip and spine has also been noted in clinical studies. However, a prospective study that correlated BMD response with expression of bone markers found that mean biomarker response was marginally larger for the BMD nonresponders at either site than for the responders.^62^ The authors concluded that the apparent failure of BMD response in certain patients receiving teriparatide may reflect a combination of measurement imprecision and variable bone remodeling balance. Finally, from studies of combination therapy, hip BMD has been noted to improve significantly when PTH is combined with bisphosphonates compared with PTH monotherapy alone.^63^ In contrast, most studies do not show a benefit of combination therapy on spine BMD.^63^

High direct cost of treatment remains one of the main drawbacks that limits the broad application of this treatment.^17^ As a result, it has been suggested from pharmacoeconomic studies that PTH treatment is only justified among patients with osteoporosis in the highest quartile of fracture risk. In this respect, strategies that can potentially reduce the cost of therapy while maintaining good efficacy should be considered and further explored.

Early results of novel formulations of teriparatide suggest that new formulations, such as intranasal and patch teriparatide administration may be safe, effective, and well‐tolerated options that could potentially improve adherence. However, further clinical studies are required to confirm this.

## Conclusion

Although there are no direct comparative studies that have been published, both teriparatide (PTH [1‐34]) and full‐length PTH (1‐84) appear to be effective for reducing fracture risk in patients with osteoporosis in a range of clinical studies and settings. Once‐daily and once‐weekly PTH also appear to have beneficial effects on bone physiology to produce clinical effects, although the intensity of dosing may influence parameters, such as bone formation markers and BMD. Clinical efficacy has been noted with different dosing strategies, although these have not been directly compared to date. Novel formulations of PTH involving less invasive routes of administration represent a potential advance in patient acceptability, although further studies are required to establish clinical efficacy and safety.

## Disclosures

SYL is an employee of Eli Lilly Korea Ltd. KMK and YR have no conflicts of interest to declare.
